# Liver status in congenital, untreated, isolated GH deficiency

**DOI:** 10.1530/EC-14-0078

**Published:** 2014-08-28

**Authors:** Rachel D C A Diniz, Renata M Souza, Roberto Salvatori, Alex Franca, Elenilde Gomes-Santos, Thiago O Ferrão, Carla R P Oliveira, João A M Santana, Francisco A Pereira, Rita A A Barbosa, Anita H O Souza, Rossana M C Pereira, Alécia A Oliveira-Santos, Allysson M P Silva, Francisco J Santana-Júnior, Eugênia H O Valença, Viviane C Campos, Manuel H Aguiar-Oliveira

**Affiliations:** Division of Endocrinology, Diabetes and Metabolism Federal University of Sergipe Aracaju Brazil; 1 Division of Endocrinology, Diabetes and Metabolism the Johns Hopkins University School of Medicine 1830 East Monument Street Suite #333, Baltimore, Maryland, 21287 USA; 2 Division of Hepatology Federal University of Sergipe Aracaju Brazil; 3 Division of Radiology Federal University of Sergipe Aracaju Brazil

**Keywords:** GH, IGF1, NAFLD, isolated GH deficiency

## Abstract

Nonalcoholic fatty liver disease (NAFLD) is known to be associated with insulin resistance, atherosclerosis, and low serum IGF1 levels. We have described a large cohort of patients with isolated GH deficiency (IGHD) due to the c.57+1G→A mutation in the GHRH receptor gene. These subjects have increased insulin sensitivity (IS), delayed atherosclerosis, and normal longevity. We hypothesized that, despite visceral obesity, NAFLD would be absent or mild due to the increased IS. To assess the prevalence and severity of NAFLD in adult subjects with lifetime, congenital, untreated IGHD, we studied 22 IGHD adults and 25 controls (COs) matched for age and sex. NAFLD was assessed by a comprehensive liver function panel, and ultrasonographic pattern (hyperechogenic pattern, HP) coded as follows: 0, absent; 1, mild; 2, moderate; and 3, severe. Compared with COs, IGHD individual had lower serum IGF1 (*P*<0.0001), higher total cholesterol (*P*=0.027), lower prothrombin time (*P*=0.017), and similar activated partial thromboplastin time and fibrinogen values. Alanine transaminase (ALT) values were similar in the two groups, but aspartate transaminase was higher in IGHD (*P*=0.013). However, more IGHD subjects (7/22) than COs (3/23) had ALT above the upper limit of normal (*P*=0.044). The prevalence of NAFLD was higher in IGHD than COs (61 vs 29%, *P*=0.032), and the HP score was higher in IGHD than COs (*P*=0.041), but severe NAFLD was not observed in any IGHD (or CO) individual. Liver HP score is increased in lifetime, untreated, congenital IGHD, but the increase in transaminases is mild, suggesting a lack of advanced forms of NAFLD.

## Introduction

Nonalcoholic fatty liver disease (NAFLD) is a manifestation of the metabolic syndrome and is associated with very common conditions such as obesity, type 2 diabetes, hypertension, dyslipidemia, and atherosclerosis (1). NAFLD includes the mere accumulation of lipid within hepatocytes (hepatic steatosis, HS), or the inflammation of the liver (nonalcoholic steatohepatitis, NASH), liver fibrosis, or cirrhosis (2). The evolution of HS to NASH occurs through the development of insulin resistance, by accumulation of fat in visceral tissue, and increased oxidative stress, with the consequent development of hepatitis (3). High leptin and low adiponectin levels have also been associated with this evolution (4). The consequences of NAFLD on mortality are an issue of debate (5), as this can be influenced by variable definitions and associated conditions. Cardiovascular mortality is increased in NASH cirrhosis compared with other types of cirrhosis, and the incidence of associated hepatocellular carcinoma is higher than 10% in 5 years (6). It is therefore important to define the causes of NAFLD.

Adult-onset GH deficiency (AOGHD) constitutes a specific model of metabolic syndrome (7, 8), with visceral obesity, insulin resistance, accelerated atherosclerosis, and increased cardiovascular mortality (9). AOGHD has been associated with NAFLD (10, 11, 12). Some of the effects of GH are mediated by circulating IGF1, which is mostly of liver origin. IGF1 circulates bound to six IGF1-binding proteins, mainly IGFBP3. Low serum levels of GH or IGF1 and IGF1/IGFBP3 ratio (reflecting low IGF1 bioavailability) have been hypothesized to contribute to NAFLD in AOGHD (13, 14). However, congenital (vs acquired) isolated GH deficiency (IGHD) may have different consequences in terms of NAFLD.

In Itabaianinha County, in the Brazil Northeast, we have identified a large cohort of patients (more than 100 over seven generations) with congenital IGHD due to a homozygous mutation (c.57+1G→A) in the GHRH receptor gene (*GHRHR*) (15). These subjects have very low circulating serum IGF1 levels and could therefore be predisposed to developing NAFLD (16). Despite abdominal obesity and unfavorable cardiovascular risk profile (high total and LDL-C and high C-reactive protein) (17, 18), they have increased insulin sensitivity (19), high adiponectin and normal leptin serum levels (20), no evidence of premature atherosclerosis (21), and normal longevity (22). As NAFLD is related to insulin resistance, we hypothesized that, despite low IGF1, NAFLD would be absent or mild in this model. The aim of this work was to assess the liver status of these subjects.

## Subjects and methods

### Subjects

This was a cross-sectional study conducted in Itabaianinha County in the Northeastern Brazilian state of Sergipe. We recruited volunteers (aged 20–59 years) through advertisement placed in the local Dwarfs Association building, and through communication with subjects. Inclusion criteria for IGHD were short stature and genotype-proven homozygosity for the c.57+1G→A *GHRHR* mutation, whereas COs were normal-statured individuals proven to be homozygous for the WT *GHRHR* allele. For both groups, exclusion criteria were a history of current or past excessive alcohol intake (defined by an average daily consumption of more than 20 g of alcohol), % fat mass below 20% and above 50%, diabetes mellitus, use of glucocorticoids, GH, and thyroid hormones, positive hepatitis B and C serology, and inherited, autoimmune, cholestatic, or drug-induced liver disease. Individuals had been previously matched for age, sex, and percentage of fat mass (assessed by DXA) (23). From the 53 IGHD adult living individuals identified previously (22), two declined, 16 were excluded by age criterion, nine by GH treatment, one for alcoholism, one because of portal hypertensions due to *Schistosoma mansoni*, and two because of fat mass lower than 20%. Therefore, 22 IGHD and 25 CO volunteers were enrolled.

The Federal University of Sergipe Institutional Review Board approved these studies, and all subjects gave written informed consent.

### Study protocol

#### Anthropometric measurement

The subjects' height and weight were measured using a wall-mounted stadiometer (Digital Wall Mounted Stadiometer, Model HM210D, Charder Medical Weighing and Measuring Products, Taichung City Taiwan, R.O.C) and scale (Charder MS2510 Platform Scale). Waist circumference was measured at half the distance between the last rib and the superior margin of iliac crest, and hip circumference was measured at the level of femoral trochanters. Height, weight, and BMI were converted to SDSs using the website http://www.phsim.man.ac.uk/SDSCalculator/SDSCalculator.aspx.

#### Sonography

Sonographic measurements were all performed by a single radiologist (T O F), using Medison Sono Ace 8000SE (Samsung, Seoul, South Korea), with a 3.5 MHz convex-array probe. The probe was touched as adjusted in such a way that the liquid contents of the gall bladder and the blood in the inferior vena cava were nonechogenic. Gain curve was adjusted to the neutral position. The patient was placed supine and the probe was placed on the right hypochondrium in the longitudinal, transverse, and oblique planes. To diagnose HS, we used a hyperechogenic pattern (HP) coded as follows: 0, absent; 1, mild (hepatic parenchyma with a subtle increase in echogenicity and sound beam attenuation and a subtle decrease in the visualization of the diaphragm and intrahepatic vascularization); 2, moderate (hepatic parenchyma with a moderate increase in echogenicity and sound beam attenuation and a moderate decrease in the visualization of the diaphragm and intrahepatic vascularization); and 3, severe (hepatic parenchyma with a increase in echogenicity and sound beam attenuation, with a marked or complete loss of the visualization of the diaphragm and of the intrahepatic vascularization) (24). The sonographer was blinded to the laboratory data, but it was impossible to blind him to the IGHD phenotype, due to the severe height reduction. One subject in each group did not undergo ultrasound imaging.

#### Laboratory analyses

Fasting blood samples were collected between 0700 and 0900 h. Alanine transaminase (ALT), aspartate transaminase (AST), gamma-glutamyl transpeptidase (GGT), total cholesterol and fractions, triglycerides, fibrinogen, prothrombin time (PT), international normalized ratio (INR), prothrombin activity (PA), and activated partial thromboplastin time (APTT) were determined by standard laboratory techniques. AST and ALT were divided by the respective upper limit (AST/upper limit and ALT/upper limit respectively). Anti-HBC IgM, HbsAg, and anti-HCV were measured by electrochemiluminescence, ELECSYS 2010 (Roche Diagnostics). IGF1 was measured by a solid-phase, enzyme-labeled chemiluminescent immunometric assay IMMULITE 2000 (Siemens Healthcare Diagnostics Products Ltd, Malvern, PA, USA), with a sensitivity of 25 ng/ml. Intra- and interassay variabilities were 3.1 and 6.1% respectively. All the tests were carried out in the Laboratory of University Hospital of the Federal University of Sergipe, in Aracaju, capital of Sergipe state.

### Statistical analysis

Data are expressed as mean±s.d. Continuous variables were compared by the independent-samples *t-*test and categorical and not normal variables (triglycerides) by the Mann–Whitney *U* test. Percentages were compared by Fisher's exact test. The SPSS/PC 15.0 software (SPSS, Inc.) was used. *P* values <0.05 were considered significant.

## Results

Demographic and anthropometric data are given in Table 1. IGHD and COs were of similar age and sex and have a similar BMI. As expected, IGHD subjects had lower height and weight, but had a higher waist/hip ratio than COs. Laboratory and sonography data are given in Table 2. IGF1 levels were extremely low in IGHD individuals, and 16 out of 22 IGHD individuals had values below the limit of sensitivity of 25 ng/ml. These values were assumed to be 25 for the purpose of the statistical analyses. IGHD subjects had higher total and LDL-C, lower PT, and similar HDL-C, triglycerides, γ-GT, APTT, and fibrinogen values compared with COs.

Average ALT values were similar in the two groups, while AST was higher in IGHD subjects. However, more IGHD subjects (7/22) than COs (3/23) had an ALT/upper limit of normal ratio above one (*P*=0.044): four IGHD subjects (1.04, 1.13, 1.29, and 1.87) and three COs (1.20, 1.33, and 1.42) less than twice, and three IGHD subjects more than twice (2.09, 2.20, and 2.67). Four IGHD subjects (1.00, 1.13, 1.82, and 1.85) and one CO individual (1.31) had an AST/upper limit ratio above one. No individual in either group had an AST/upper limit ratio higher than two. While the average HP score was higher in IGHD subjects than COs (*P*=0.041), severe NAFLD was not observed in any IGHD (or CO) individual. It has been observed that 61% of IGHD subjects (13/21) had HS (ten mild and three moderate), while only 29% of COs (7/24) had HS (five mild and two moderate) (*P*<0.032).

## Discussion

NAFLD is a very important public health care problem, linked to several common conditions such as visceral obesity, diabetes, atherosclerosis, and increased cardiovascular mortality. NAFLD can evolve to NASH, liver fibrosis, and cirrhosis and, eventually, to liver failure, becoming an important cause of liver transplantation (4).

Our data show that NAFLD is more frequent (although sonographic and biochemically mild) in individuals with severe congenital IGHD than in indigenous controls, matched by fat mass % and BMI, but with higher WHR. Such increase in WHR is due to a specific increase in visceral adiposity (23).

HS, together with insulin resistance, is correlated with downregulation of the GH signaling. The epidermal growth factor receptor (EGFR) signaling system is a key player in all stages of the liver response to injury, from early inflammation and hepatocellular proliferation to fibrogenesis and neoplastic transformation (24). The EGFR receptor heterodimerizes with the IGF1 receptor, playing major roles in liver responses to injury (24). The GH/EGFR pathway downregulation is probably the mechanism responsible for liver regeneration deficiency associated with HS, which can be partially rescued in *ob*/*ob* mice by GH administration (25). Although GH treatment has a positive regenerative effect on the liver after hepatic resection (25), its efficacy in reducing NAFLD in hypopituitarism is controversial, with positive (26, 27) and negative (28) reports. Furthermore, IGF1 treatment does not improve NAFLD in individuals with Laron dwarfism, a known model of GH resistance (29).

Hypopituitarism (often with GHD) has been associated with NAFLD (30), but it is not clear whether NAFLD is a consequence of GHD (and IGF1 deficiency), or whether they are both unrelated consequences of hypopituitarism. It has been shown, using liver biopsy and the hyaluronic acid as a fibrosis marker, that GH may be involved in the mechanism of triglyceride secretion from hepatocytes. Therefore, low levels of IGF1 and low IGF1/IGFBP3 ratio (reflecting low IGF1 bioavailability) may be associated with the accumulation of lipids and advanced fibrosis in NAFLD (14). We have previously shown that IGHD individuals from the Itabaianinha cohort have very low serum GH and IGF1 levels, but have a high total IGF1+IGF2/IGFBP3 molar ratio (due to a compensatory increase in IGF2/IGFBP3 molar ratio) (16). We therefore hypothesize that this high IGF1+IGF2/IGFBP3 molar ratio, resulting in high total (IGF1+IGF2) bioavailability, could slow down the evolution form HS to fibrosis or cirrhosis.

As a whole, IGHD subjects have higher serum AST, and the number of individuals with ALT above the normal values was higher in IGHD than COs. The lower PT may indicate higher synthesis of prothrombin by the liver, without obvious clinical relevance. Therefore, we conclude that, while IGHD subjects have more NAFLD than controls, there is no evolution to severe stages of NAFLD. Although we did not perform liver biopsies, the gold standard method to diagnose NAFLD, a comprehensive biochemical and clotting liver evaluation, supports our conclusion of a mild NAFLD pattern in these IGHD subjects. The HS frequency of our IGHD subjects (61%) was similar to that reported (55%, 6/11) in adult patients with Laron dwarfism (GH resistance). The severity of HS was also similar (mild in 48% of our IGHD and in 45% of Laron dwarfs). Interestingly, because the presence of NAFLD was found not to correlate with age, sex, degree of obesity, blood lipids, HOMA-IR, and therapy by statins or IGF1, the data were reported by Prof. Laron to be ‘not fitting with present theories of the development of fatty liver (29)’. Our data agree with their conclusion on the difficulty in differentiating between the influences of longstanding GH/IGF1 deficiency and other factors on the development of NAFLD.

Among patients with hypothalamic and pituitary dysfunction, NAFLD was diagnosed with a median of 3 years after the diagnosis of pituitary dysfunction, with a high prevalence of cirrhosis and liver-related death (4). Given the relatively young age of the subjects studied in this work, one possibility is that such progression in congenital IGHD may occur later in life. However, having worked with this cohort (including providing primary medical care) for more than 20 years, we did not observe any case of clinical liver disease except one portal fibrosis due to *S. mansoni* infection. Furthermore, no liver disease was reported in death certificates of 22 IGHD deceased individuals (22).

The causes of this lack of progression may be multiple. In addition to the high IGF1+IGF2/IGFBP3 ratio, other protective factors may have a role. One of them is the high serum adiponectin level (19), as low adiponectin levels are classically associated with insulin resistance. A second protective factor is the normal leptin (19). Lack of leptin signaling in leptin-deficient and -resistant mice is associated with NAFLD (31). The normal leptin serum values of these IGHD individuals suggest normal leptin signaling.

In conclusion, the prevalence of NAFLD and the liver HP score are increased in adults with congenital, lifetime, untreated IGHD, but with only a modest increase in transaminases, suggesting the lack of advanced forms of NAFLD. This finding contrasts with the association of severe forms of NAFLD in acquired hypopituitarism and weakens the hypothesis of a causal relationship of GH/IGF1 deficiency in the pathogenesis of NAFLD.

## Figures and Tables

**Table 1:** Demographic and anthropometric measurements in isolated GH-deficient IGHD (*n*=22) and control groups, COs (*n*=25). Data are expressed as mean±s.d., except for sex.

	**IGHD**	**CO**	***P***
Age (years)	39.3±12.0	37.8±10.9	0.652
Sex	11 M	10 M	0.595
Height (m)	1.3±0.1	1.6±0.1	<0.0001
SDS height/age	−5.8±1.5	−0.8±0.8	<0.0001
Weight (kg)	39.3±7.8	67.4±13.1	<0.0001
BMI (kg/m^2^)	24.0±5.0	25.3±4.2	0.329
Waist (cm)	76.6±10.0	84.8±9.9	0.008
Hip (cm)	77.6±9.0	93.6±8.9	<0.0001
Waist/hip ratio	0.98±0.1	0.90±0.1	<0.0001

**Table 2:** Laboratorial data in isolated GH-deficient IGHD (*n*=22) and control groups, COs (*n*=25). Data are expressed as mean±s.d.

	**IGHD**	**CO**	***P***
IGF1	33.4±20.7	160.6±51.9	<0.0001
Total cholesterol (mg/dl)	200.4±51.2	171.6±28.1	0.027
LDL-C (mg/dl)	124.9±40.5	102.9±24.2	0.032
HDL-C (mg/dl)	53.0±12.1	48.7±11.3	0.220
Triglycerides (mg/dl)	113.2±100.0	98.1±63.6	0.551
AST (U/l)	32.6±14.1	24.1±7.1	0.013
ALT (U/l)	45.3±28.5	34.4±11.5	0.106
AST/upper limit	0.8±0.4	0.6±0.2	0.013
ALT/upper limit	1.0±0.6	0.8±0.3	0.106
GGT (U/l)	42.2±37.5	28.9±18.7	0.144
PT (s)	10.7±0.7	11.2±0.4	0.017
PA (%)	98.1±4.8	97.7±4.3	0.793
INR (%)	1.0±0.0	1.0±0.0	0.216
APTT (s)	26.8±2.8	26.8±1.4	0.996
Fibrinogen (mg/dl)	290.7±46.8	272.6±63.3	0.292
HP	0.8±0.7	0.4±0.7	0.041

PT, prothrombin time; PA, prothrombin activity; APTT, activated partial thromboplastin time; HP, hyperechogenic pattern: 0, absent; 1, mild, 2, moderate and 3, severe.

## References

[1] Villanova N, Moscatiello S, Ramilli S, Bugianesi E, Magalotti D, Vanni E, Zoli M, Marchesini G (2005). Endothelial dysfunction and cardiovascular risk profile in nonalcoholic fatty liver disease. Hepatology.

[2] Levene AP, Goldin RD (2012). The epidemiology, pathogenesis and histopathology of fatty liver disease. Histopathology.

[3] Adams LA, Angulo P (2005). Recent concepts in non-alcoholic fatty liver disease. Diabetic Medicine.

[4] Adams LA, Feldstein A, Lindor KD, Angulo P (2004). Nonalcoholic fatty liver disease among patients with hypothalamic and pituitary dysfunction. Hepatology.

[5] Ong JP, Pitts A, Younossi ZM (2008). Increased overall mortality and liver-related mortality in non-alcoholic fatty liver disease. Journal of Hepatology.

[6] Yatsuji S, Hashimoto E, Tobari M, Taniai M, Tokushige K, Shiratori K (2009). Clinical features and outcomes of cirrhosis due to non-alcoholic steatohepatitis compared with cirrhosis caused by chronic hepatitis C. Journal of Gastroenterology and Hepatology.

[7] Attanasio AF, Mo D, Erfurth EM, Tan M, Ho KY, Kleinberg D, Zimmermann AG, Chanson P (2010). Prevalence of metabolic syndrome in adult hypopituitary growth hormone (GH)-deficient patients before and after GH replacement. Journal of Clinical Endocrinology and Metabolism.

[8] Di Somma C, Pivonello R, Pizza G, De Rosa A, Lombardi G, Colao A, Savastano S (2010). Prevalence of the metabolic syndrome in moderately–severely obese subjects with and without growth hormone deficiency. Journal of Endocrinological Investigation.

[9] Rosen T, Bengtsson BA (1990). Premature mortality due to cardiovascular disease in hypopituitarism. Lancet.

[10] Ichikawa T, Hamasaki K, Ishikawa H, Ejima E, Eguchi K, Nakao K (2003). Non-alcoholic steatohepatitis and hepatic steatosis in patients with adult onset growth hormone deficiency. Gut.

[11] Tarantino G, Savastano S, Colao A (2010). Hepatic steatosis, low-grade chronic inflammation and hormone/growth factor/adipokine imbalance. World Journal of Gastroenterology.

[12] Xu L, Xu C, Yu C, Miao M, Zhang X, Zhu Z, Ding X, Li Y (2012). Association between serum growth hormone levels and nonalcoholic fatty liver disease: across-sectional study. PLoS ONE.

[13] Volzke H, Nauck M, Rettig R, Dorr M, Higham C, Brabant G, Wallaschofski H (2009). Association between hepatic steatosis and serum IGF1 and IGFBP-3 levels in a population-based sample. European Journal of Endocrinology.

[14] Ichikawa T, Nakao K, Hamasaki K, Furukawa R, Tsuruta S, Ueda Y, Taura N, Shibata H, Fujimoto M, Toriyama K (2007). Role of growth hormone, insulin-like growth factor 1 and insulin-like growth factor-binding protein 3 in development of non-alcoholic fatty liver disease. Hepatology International.

[15] Salvatori R, Hayashida CY, Aguiar-Oliveira MH, Phillips JA, Souza AH, Gondo RG, Toledo SP, Conceição MM, Prince M, Maheshwari HG (1999). Familial dwarfism due to a novel mutation of the growth hormone-releasing hormone receptor gene. Journal of Clinical Endocrinology and Metabolism.

[16] Aguiar-Oliveira MH, Gill MS, Barretto ESA, Alcântara MR, Miraki-Moud F, Menezes CA, Martinelli CE, Pereira FA, Salvatori R, Levine MA (1999). Effect of severe growth hormone (GH) deficiency due to a mutation in the GH releasing hormone receptor on insulin-like growth factors (IGF-1), IGF-binding proteins, and ternary complex formation throughout life. Journal of Clinical Endocrinology and Metabolism.

[17] Barretto ESA, Gill MS, Freitas ME, Magalhães MM, Souza AH, Aguiar-Oliveira MH, Clayton PE (1999). Serum leptin and body composition in children with familial GH deficiency (GHD) due to a mutation in the growth hormone-releasing hormone (GHRH) receptor. Clinical Endocrinology.

[18] Barreto-Filho JA, Alcântara MR, Salvatori R, Barreto MA, Sousa AC, Bastos V, Souza AH, Pereira RM, Clayton PE, Gill MS (2002). Familial isolated growth hormone deficiency is associated with increased systolic blood pressure, central obesity, and dyslipidemia. Journal of Clinical Endocrinology and Metabolism.

[19] Oliveira CR, Salvatori R, Barreto-Filho J, Rocha IE, Mari A, Pereira RM, Campos VC, Menezes M, Gomes E, Meneguz-Moreno RA (2012). Insulin sensitivity and β-cell function in adults with lifetime, untreated isolated growth hormone deficiency. Journal of Clinical Endocrinology and Metabolism.

[20] Oliveira CR, Salvatori R, Meneguz-Moreno RA, Aguiar-Oliveira MH, Pereira RM, Valença EH, Araujo VP, Farias NT, Silveira DC, Vieira JG (2010). Adipokine profile and urinary albumin excretion in isolated growth hormone deficiency. Journal of Clinical Endocrinology and Metabolism.

[21] Oliveira JL, Marques-Santos C, Barreto-Filho JA, Ximenes-Filho R, Britto AV, Souza AH, Prado CM, Oliveira CR, Pereira RM, Vicente TA (2006). Lack of evidence of premature atherosclerosis in untreated severe isolated growth hormone (GH) deficiency due to a GH-releasing hormone receptor mutation. Journal of Clinical Endocrinology and Metabolism.

[22] Aguiar-Oliveira MH, Oliveira FT, Pereira RM, Oliveira CR, Blackford A, Valenca EH, Santos EG, Gois-Junior MB, Meneguz-Moreno RA, Araujo VP (2010). Longevity in untreated congenital growth hormone deficiency due to a homozygous mutation in the GHRH receptor gene. Journal of Clinical Endocrinology and Metabolism.

[23] Gomes-Santos EG, Salvatori R, Ferrão TO, Oliveira CR, Diniz RD, Santana JA, Pereira FA, Barbosa RA, Souza AH, Melo EV (2014). Increased visceral adiposity and cortisol to cortisone ratio in adults with congenital lifetime isolated GH deficiency. Journal of Clinical Endocrinology and Metabolism.

[24] Berasain C, Avila MA (2014). The EGFR signalling system in the liver: from hepatoprotection to hepatocarcinogenesis. Gastroenterology.

[25] Collin de l'Hortet A, Zerrad-Saadi A, Prip-Buus C, Fauveau V, Helmy N, Ziol M, Vons C, Billot K, Baud V, Gilgenkrantz H (2014). GH administration rescues fatty liver regeneration impairment by restoring GH/EGFR pathway deficiency. Endocrinology.

[26] Takahashi Y, Iida K, Takahashi K, Yoshioka S, Fukuoka H, Takeno R, Chihara K (2007). Growth hormone reverses nonalcoholic steatohepatitis in a patient with adult growth hormone deficiency. Gastroenterology.

[27] Nishizawa H, Iguchi G, Murawaki A, Fukuoka H, Hayashi Y, Kaji H, Takahashi Y (2012). Nonalcoholic fatty liver disease in adult hypopituitary patients with GH deficiency and the impact of GH replacement therapy. European Journal of Endocrinology.

[28] Gardner CJ, Irwin A, Daousi C, Macfarlane I, Joseph F, Bell J, Thomas EL, Adams V, Kemp G, Cuthbertson DJ (2012). Nonalcoholic fatty liver disease, growth hormone deficiency and the effects of growth hormone replacement: a Liverpool magnetic resonance spectroscopy study. European Journal of Endocrinology.

[29] Laron Z, Laron Z, Kopchick JJ (2011). Nonalcoholic fatty liver disease (NaFLD) in patients with Laron syndrome. Laron Syndrome-From Man to Mouse: Lessons from Clinical and Experimental Experience.

[30] Nyenwe EA, Williamson-Baddorf S, Waters B, Wan JY, Solomon SS (2009). Non-alcoholic fatty liver disease and metabolic syndrome in hypopituitary patients. American Journal of the Medical Sciences.

[31] Koteish A, Diehl AM (2001). Animal models of steatosis. Seminars in Liver Disease.

